# The clinicopathologic and immunohistochemical features of 60 cutaneous glomus tumor: a retrospective case series study^☆^

**DOI:** 10.1016/j.abd.2023.03.005

**Published:** 2023-11-23

**Authors:** Yuehua Sun, Ruiqun Qi, Ze Wu, Xiaodong Zhang, Jun Niu

**Affiliations:** a Department of Dermatology, General Hospital of Northern Theater Command, Shenyang, China; b Key Laboratory of Immunodermatology, Ministry of Education, NHC, National and Local Joint Engineering Research Center of Immunodermatological Theranostics, The First Hospital of China Medical University, Shenyang, China

**Keywords:** Calcitonin gene-related peptide, Glomus tumor, Interleukin-1beta, Interleukin-6, Pain

## Abstract

**Background:**

Glomus Tumor (GT) are benign neoplasms that originate from mesenchymal cells. It presents as tenderness and cold allodynia in the digits, especially in the subungual region. There are a few studies that investigated the mechanism of pain.

**Objectives:**

To analyze the clinical-pathologic characteristics of GT and to identify the expression of IL-1β, IL-6, and CGRP in it, further, to explore the possible mechanism of pain.

**Methods:**

The clinical and pathological data of 60 GT patients were retrospectively analyzed. Tissue microarrays and immunohistochemistry were used to measure the expression of IL-1β, IL-6 and CGRP.

**Results:**

GT is more common in females and the ratio of male to was near to 1:2, mostly in middle-aged people. It often occurs in fingertips, especially the thumbs. Patients often present with spontaneous pain, tenderness, and cold hypersensitivity. Both the two pain mediators IL-1β and IL-6 were highly expressed in GT cells of patients with and without cold hypersensitivity. While CGRP was not expressed in GT.

**Study limitations:**

Low sample size and further research is needed to explore the specific mechanism.

**Conclusions:**

IL-1β and IL-6 were highly expressed in GT cells, suggesting that IL-1β and IL-6 have certain nociceptive roles in GT. In the 4 patients with cold intolerance, the intensity of IL-1β and IL-6 staining was also strong, suggesting that they may not play a role in the cold hypersensitivity. However, since there are only 4 patients with cold intolerance, it’s necessary to conduct further in-depth research using a larger sample size. The specific role of CGRP in GT has not been found yet.

## Introduction

Glomus Tumor (GT) is a benign neoplasm of mesenchymal origin tumor arising from the glomus body. It generally occurs in the fingers, especially the nailbeds.^1^ GT is often misdiagnosed, leading to pain and delay in treatment. Allodynia, localized tenderness, and cold hypersensitivity are the key to diagnosing GT. However, patients do not always show these symptoms simultaneously in practice. Although previous studies showed that nerve fibers, mast cells and cyclooxygenase-2 are abundant in GT,^2^, ^3^, ^4^ an understanding of pain mechanisms is still incomplete. In this study, a retrospective case series study of 60 patients with GT was performed. The authors examined the expression of Interleukin-1Beta (IL-1β), Interleukin-6 (IL-6), and Calcitonin Gene Related Peptide (CGRP) in GT tissues and analyzed their possible role in GT.

## Materials and methods

### Samples collection of glomus tumor

The data of 60 GT cases were collected, all of whom visited the General Hospital of Northern Theater Command from 2002 to 2019 and were diagnosed as GT by routine pathological examination (Hematoxylin and eosin staining). The clinical data were retrieved from the electronic case system. All cases were reconfirmed by two dermatopathologists. There are three types of GT based on the proportions of glomus cells, vascular structures, and smooth muscle: solid glomus tumor, glomangioma, and glomangiomyoma.^5^

### Tissue microarrays (TMA)

Paraffin blocks and sections of the 60 GT samples were collected and were assessed microscopically. A 1‒2 mm point within each section was selected, and a semi-automatic tissue drill was used to isolate a tissue cylinder (1‒2 mm in diameter) or in the center of the donor block, where glomus tumor cells were intensively located. These isolated samples were then inserted into 1 new paraffin block for TMA. TMA (3 μm thickness) was sliced with an automatic paraffin section system. Immunohistochemistry was performed after sectioning.

### Immunostaining

All pathological samples were sectioned. The sections were deparaffinized and rehydrated using xylene and graded alcohol. The sections were then blocked with 2% bovine serum albumin, incubated with IL-1 Beta Polyclonal Rabbit Polyclonal Antibody (China, Servicebio,16806-1-AP) at a dilution of 1:200; Anti-IL-6 Rabbit pAb (China, Servicebio,Gb1117) at a dilution of 1:800; Anti-CGRP antibody (China, Servicebio, 16630-1-AP) at a dilution of 1:200 for 2 hours, followed by incubation with biotinylated secondary antibodies for 1.5 hours and then horseradish peroxidase-bound streptavidin. Subsequently, 3-amino-9-ethylcarbazole was added dropwise over 3‒5 minutes, and sections were counterstained with hematoxylin.

### Sample assessment

Immunohistochemical results of IL-1β, IL-6 and CGRP in GT were recorded to determine the differences between the groups. The three tissue chips were electronically scanned, observed, and analyzed using CaseViewer. IL-1β- and IL-6-positive staining was cytoplasmic. The results were expressed by combining the dyeing strength and percent as described.^6^ The dyeing strength was scored as follows: 0‒no staining, 1‒weak staining, 2‒medium staining, and 3‒strong staining. The dyeing percent was scored as follows: 0-point: 0% to 5% of the total cells were stained; 1-point: 6% to 25% staining; 2-points: 26% to 50% staining; 3-points: 51% to 75% staining, and 4-points: >75%. Results were presented as based upon the product of staining intensity and percent: 0 ‒ negative (-); 1 to 7 – weakly positive (+); 8 to 10 – medium positive (++); and 11 to 12 – strongly positive (+++).

### Statistical analysis

Analysis was performed using IBM SPSS 26 program (SPSS Inc., Chicago, IL). Statistical tests included Mann-Whitney *U* test. A p<0.05 was required for the results to be considered statistically significant.

## Results

### Clinicopathologic characteristics of 60 GTs (Table 1)

#### Pathological morphology of GT

**Figure 1:**
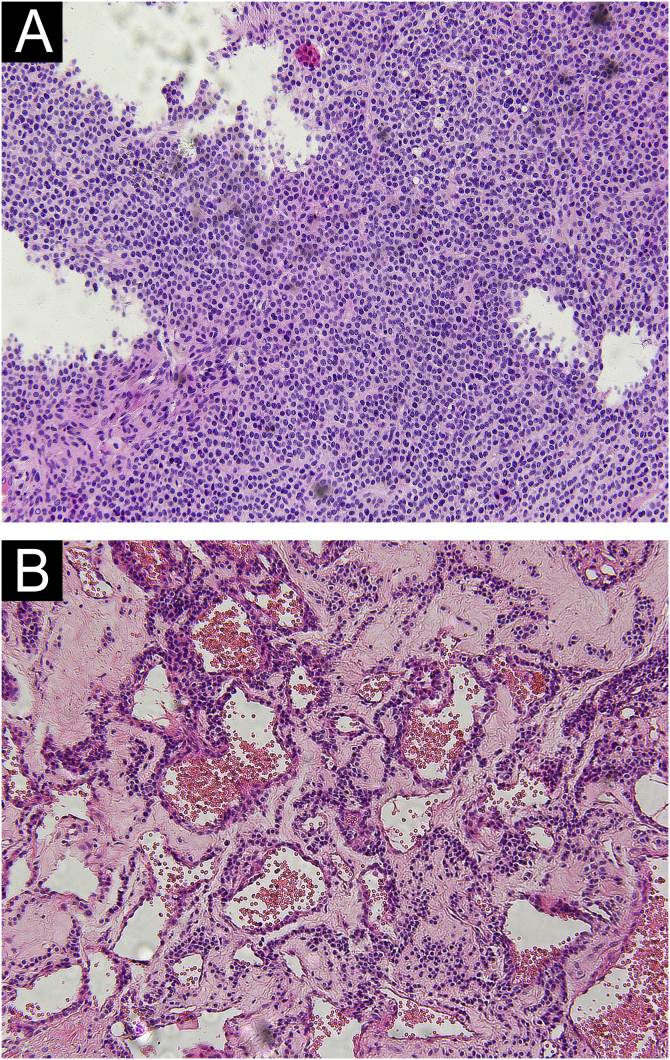
Light Microscopy of glomus tumor. (A) Solid glomus tumor was composed of monotonous round cells in solid sheets. (B) Glomangioma showed a large number of vascular lumens, which were surrounded by several layers of glomus cells. (Hematoxylin & eosin, ×100).

Different pathological forms of GT contain glomus cells, vascular lumen, and vascular smooth muscle cells, but the proportion is different. Solid glomus tumor is mostly composed of glomus cells, glomangioma is composed of glomus cells surrounding a large number of blood vessels, and a large number of vascular smooth muscle cells can be seen in glomangiomyoma. In this study, only two pathological types of solid glomus tumor and glomangioma were found (Fig. 1).

**Table 1:** Summary of clinicopathologic findings in 60 patients with glomus tumor.

Characteristics	Value
Gender, n	
Male:Female	21:39
Age at diagnosis, years	
Mean (Range)	40 (8‒92)
Duration, years	
Mean (Range)	6.31 (10 days‒30 years)
Location, n	
Digit, Extradigit	38:22
Hands and toes	40
Arms and legs	13
Trunk	6
Face	1
Left hand, right hand	21:17
Thumb	8:6
Index finger	2:3
Middle finger	4:3
Index finger	3:2
Ring finger	3:2
Palm	1:1
Under nail, Skin	21:39
Size, cm	
Mean (Range)	0.79 (0.1‒3.3)
Symptom, n	
Pain (Cold intolerance)	58 (4)
Painless	2
Pathology, n	
Solid glomus tumor	33
Glomangioma	27
Glomangiomyoma	0
Initial diagnosis, n (%)	
Glomus tumor	38 (63.3%)
Vascular tumor	8 (13.3%)
Cystis	6 (10%)
Swelling	3 (5%)
Pyogenic granuloma	2 (3.3%)
Fibroma	2 (3.3%)
Foreign body	1 (1.7%)

### Expression of IL-1β, IL-6 and CGRP in GT

**Figure 2:**
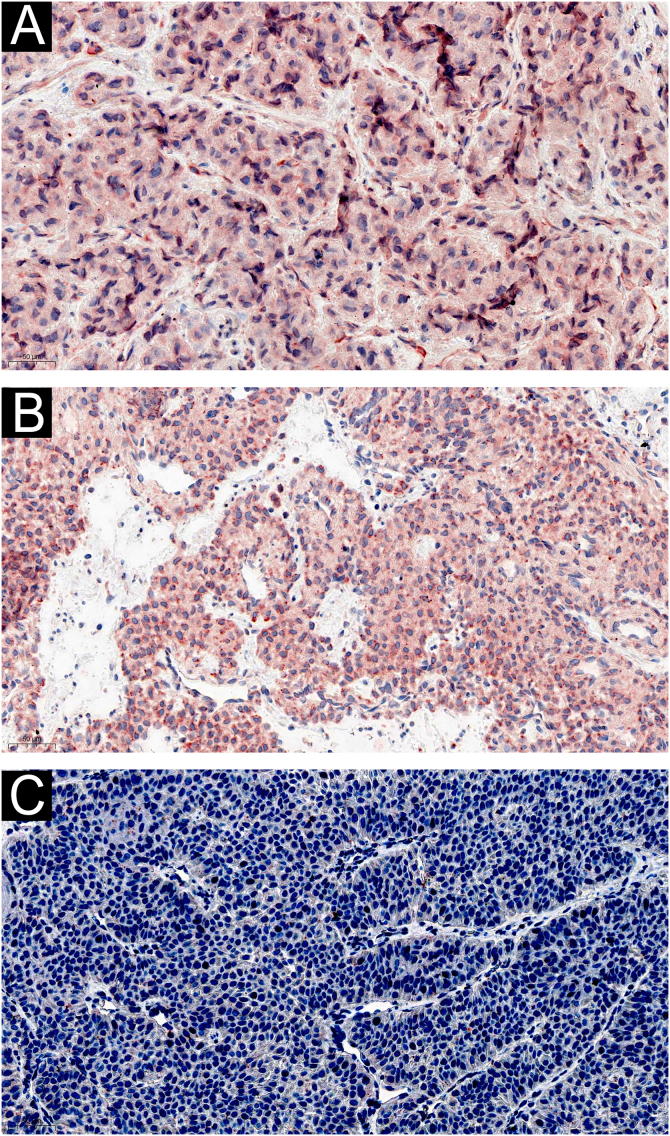
Representative images of immunohistochemical staining from glomus tumor samples. (A) IL-1β was strongly expressed in glomus cells (×200); (B) IL-6 was strongly expressed in glomus cells (×200); (C) CGRP was not expressed in glomus cells (×200).

Immunohistochemically, tumor cells in all 60 cases showed diffuse immunostaining for IL-1β and IL-6 (Fig. 2 A‒B). And there was no noticeable difference in staining intensity between IL-1β and IL-6 (*Z* = -0.750, p = 0.492). There was no difference in the expression of IL-1β in different histological types of GT (*Z* = -1.567, p = 0.155), and neither was IL-6 (*Z* = -0.685, p = 0.452). CGRP was not expressed in GT (Fig. 2C).

**Figure 3:**
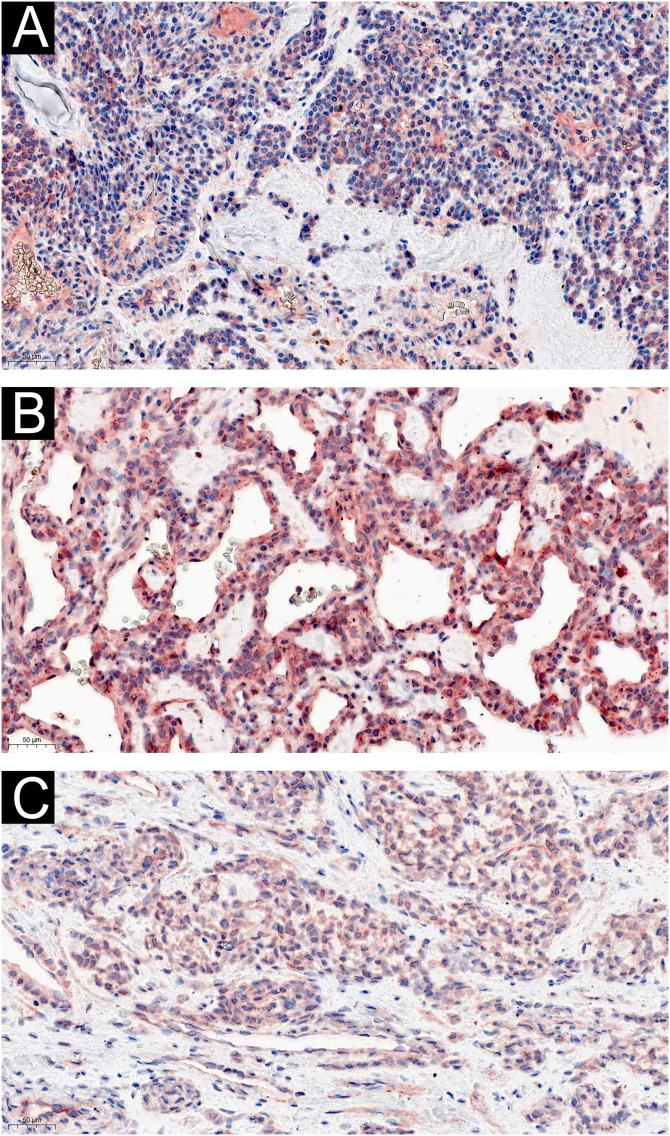
IL-1β expression: (A) moderate expression in asymptomatic glomus cells (×200); (B) strong expression in allodynia glomus cells (×200); (C) moderate expression in cold-sensitive glomus cells (×200).

**Figure 4:**
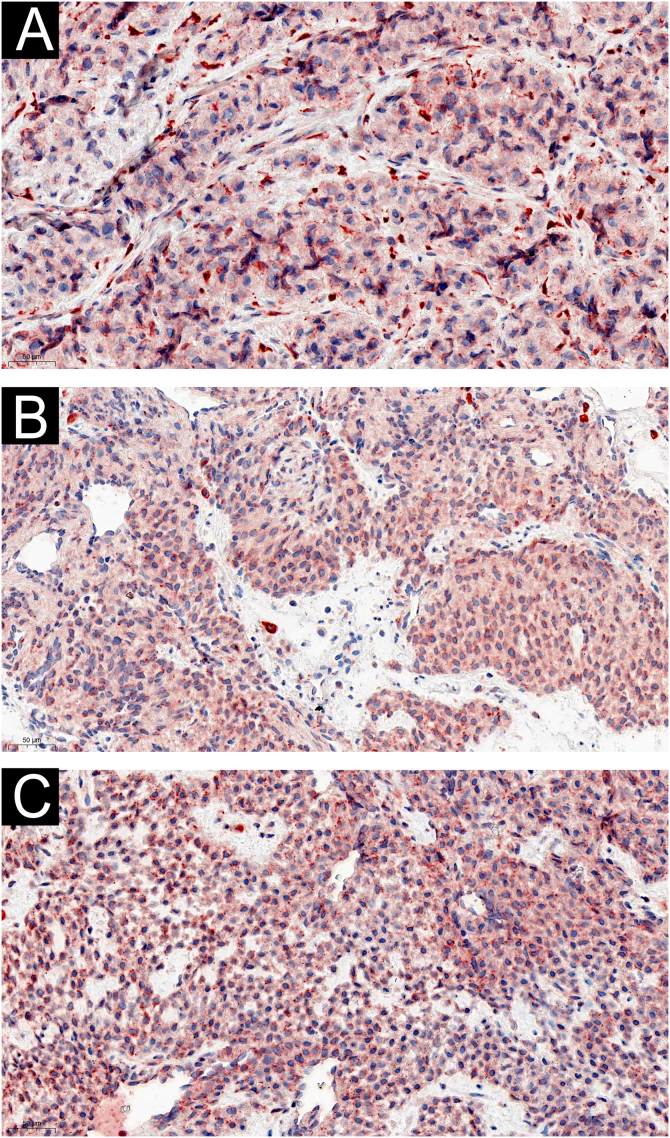
IL-6 expression: (A) strong expression in asymptomatic glomus cells (×200); (B) strong expression in allodynia glomus cells (×200); (C) strong expression in cold-sensitive glomus cells (×200).

Two of the 60 GT patients were asymptomatic, and the expression of IL-1β (Fig. 3) and IL-6 (Fig. 4) were moderately or strongly positive. Four patients who complained of cold sensitivity had strongly positive diffuse staining for IL-1β (Fig. 3) and IL-6 (Fig. 4). The expression of IL-1β did not differ between the pain cases with or without cold intolerance (Z = -0.989, p = 0.580). Neither did IL-6 (Z = -0.878, p = 0.609).

## Discussion

Current studies on GT mostly were case reports. The present study describes the clinicopathologic features by a retrospective case series study of 60 GT. There were more female patients, and the ratio of males to was near 1:2 (21:39), which was consistent with previous research.^7^ GT is more common in young and middle-aged people, and they usually have longer disease duration. All patients in this study had a single lesion. More than half of 60 cases were located in digits, especially the thumbs of both hands. The distal phalanx is the most common painful localization.^8^ The glomus body is a thermoregulatory neurovascular structure. So, when the temperature changes, the intracapsular pressure of the tumor changes and causes pain. Clinically, GT may occur in other organs such as the stomach,^9^ kidney,^10^ segmental bronchus^11^ and so on. Although rare, the number of reports has grown in recent years. The etiology of GT is unknown, but there were researchers pointed out neurofibromatosis type 1 patients are often accompanied by GT, and neurofibromatosis type 1 mutations on both alleles were found in glomus cells.^12^ There were no cases of neurofibromatosis in this study, and no other concomitant-specific diseases were found. The mean size of the tumor is 0.79 cm, the maximum tumor length is 3 cm.GT is usually benign and would not continue to increase in size indefinitely. 58 cases exhibited pain, and only 2 cases were asymptomatic, this may be due to individual differences, as each person has a different perception and threshold for pain. The majority of pathological types were solid glomus tumor and glomangioma, there was no glomangiomyoma, it was consistent with the previous study.^13^ The initial diagnosis included glomus tumor, vascular tumor, cysts and swelling, the rate of correct diagnoses was 63.3%.

Pinpoint tenderness, severe pain, and cold hypersensitivity are typical clinical manifestations of GT, although they may not be present at the same time. Few research explored the mechanism of pain in GT. 2, 14 The typical pain triad of GT is like that of neuropathic pain: spontaneous pain, hyperalgesia, and evoked pain (particularly to light touch and cold).^15^ The authors believe that the pain caused by GT is neuropathic pain. The mechanism of neuropathic pain is complex, involving central sensitization and peripheral sensitization. IL-1β and IL-6 are two kinds of pain mediators.

Previous studies showed that IL-1β could induce neuropathic pain and inflammation,^16^ and reducing its expression can reduce pain.^17^ In the red nucleus, IL-6 mediates neuropathic pain by inducing the production of TNF-α and IL-1β. IL-6 induces the production of IL-1β through the JAK2/STAT3 and ERK pathways. Neuronal pain or pain after nerve injury heavily depends on cytokine signal transduction (particularly IL-1β). The intrathecal concentration of IL-1β correlated with the degree of abnormal mechanical pain.^18^ IL-1β itself, via auto-stimulation in endothelial cells, is the mechanism of sustained IL-1β overexpression.^19^ GT is formed by the pathological proliferation of the glomus body, which is composed of endothelial cells, therefore, IL-1β may be related to the pathogenesis of GT.

IL-6 is upregulated in various preclinical models of neuropathic pain, including peripheral injury, cancer pain, and diabetic peripheral neuropathy. IL-6 levels in patients with herpes zoster are significantly increased, related to pain severity.^20^ After nerve injury, IL-6 levels are associated with allodynia due to the increased synthesis of neurons or immune cells. IL-6 interacts with neurons in the pain pathway and induces excessive excitability of nociceptors.^21^, ^22^

The neuropeptide CGRP is the principal neurotransmitter of class C nerve fiber, serving an essential role in initiating and maintaining neuropathic pain.^23^ The present study found that CGRP was not expressed in GT, suggesting that CGRP may not play a significant role in GT-related pain. The release or expression of CGRP is one of the key factors in pain. CGRP concentrations in plasma, synovial fluid, and cerebrospinal fluid are elevated during pain,^24^ but the expression of CGRP in tissues has been poorly studied and the exact mechanism has not been fully described. There are no studies on the correlation between CGRP and GT. The role of CGRP in pain is more reflected in diseases such as migraines.^25^ GT is a small local superficial tumor, and the specific role of CGRP in different diseases may be different.

The authors found that expression of IL-1β and IL-6 was high in GT cells, suggesting that IL-1β and IL-6 have certain nociceptive roles in GT. Pinpoint tenderness and severe pain often coexist. In the four patients with cold intolerance, the intensity of IL-1β and IL-6 staining was also strong, suggesting that they may not play a role in the cold hypersensitivity. However, since there are only 4 patients with cold intolerance, it’s necessary to research further in a larger sample size.

## Conclusions

GT is rare in clinics and is more common in women. Its appearance is sometimes difficult to distinguish from hemangioma or epidermal cyst. Diagnosis should be based on clinical manifestations combined with histopathological examination. Most patients are accompanied by allodynia, and a few patients have cold-sensitive pain.IL-1β and IL-6 were expressed in all GT cases, suggesting that they might participate in the pain mechanism, but not the cause of cold hypersensitivity. CGRP has not been found to play any role in it yet.

## Financial support

This work was supported by the 10.13039/501100001809National Natural Science Foundation of China (21874014).

## Author’s contribution

Yuehua Sun: Data collection, analysis and interpretation, Preparation and writing of the manuscript, Statistical analysis, Approval of the final version of the manuscript

Ruiqun Qi: Effective participation in research orientation, Study conception and planning, Approval of the final version of the manuscript

Ze Wu: Critical literature review, Approval of the final version of the manuscript

Xiaodong Zhang: Intellectual participation in propaedeutic and/or therapeutic management of studied cases, Manuscript critical review, Approval of the final version of the manuscript

Jun Niu: Approval of the final version of the manuscript.

## Conflicts of interest

None declared.
